# Enhancing preclinical dental education through a VR-based digital platform for inlay tooth preparation

**DOI:** 10.1186/s12909-025-08138-y

**Published:** 2025-11-21

**Authors:** Jiyu Sun, Xidan Zhang, Ziqi Qin, Zihua Tang, Yifeng Ruan, Yifei Shen, Zhuoli Zhu, Haiyang Yu, Jiefei Shen, Xueqi Gan

**Affiliations:** State Key Laboratory of Oral Diseases and National Clinical Research Center for Oral Diseases, West China Hospital of Stomatology, Sichuan University, No.14, 3Rd Section of Ren Min Nan Rd, Chengdu, Sichuan 610041 China

**Keywords:** Dental education, Tooth preparation, Inlays, Digital teaching platform, Virtual reality

## Abstract

**Background:**

While virtual reality (VR) is increasingly applied in dental education, most existing VR simulation platforms focus predominantly on full crown preparation. In contrast, inlay/onlay training—despite its clinical significance—remains underrepresented. This study assesses a three-dimension (3D) digital platform’s impact on teaching effectiveness and student satisfaction in preclinical dental restoration.

**Methods:**

We developed an innovative 3D digital learning platform to support preclinical dental students in performing restorative experiments. A total of 166 students voluntarily participated in the training. At the conclusion of the training, participants completed a comprehensive examination (maximum score: 60), designed to evaluate their understanding of key restorative concepts—including inlay preparation techniques, material selection, preparation design, and treatment planning. We also employed detailed questionnaires to evaluate effectiveness, user satisfaction, and willingness to continue using the platform. Test and questionnaire were specifically developed for this study and have not been published previously.

**Results:**

Of the 166 enrolled students, 152 completed the assessment, yielding a dropout rate of 8.43%. The average score in the final exam was 43.32 ± 16.17, with 48.03% of students scoring 50 or higher. Feedback revealed high satisfaction with the platform’s intuitive design and educational resources. Exploratory factor analysis identified three primary components: perceived practicality, future application prospects, and time efficiency. Most students reported increased motivation for independent study and improved understanding of fundamental inlay/onlay concepts.

**Conclusion:**

The application of a digital inlay platform appears to improve the quality of teaching in dental restoration, suggesting a promising approach for practical teaching in tooth preparation.

**Supplementary Information:**

The online version contains supplementary material available at 10.1186/s12909-025-08138-y.

## Background

With the advancement of information technology, the field of dental education is undergoing continuous transformation, gradually transitioning from the traditional teacher-centered offline teaching model to a problem-oriented, student-centric model that combines online and offline teaching. In recent years, the advent of remote teaching tools such as virtual reality (VR) technology, augmented reality (AR) technology, and online collaboration platforms has presented novel prospects for dental education [1]. Digital educational platforms foster the cultivation of students’ capacities for self-directed learning, independent thinking, and self-management - essential qualities for nurturing lifelong learners within the realm of dental professionals [2, 3].

Fixed prosthodontics is a cornerstone of restorative dentistry, but traditional teaching methods face significant practical challenges. A key objective of the preclinical prosthodontics curriculum is to familiarize students with the laboratory and clinical techniques essential for fabricating fixed restoration, including crowns and inlay/onlay, while offering structured opportunities for deliberate, skill-based practice in a controlled preclinical environment [4]. However, two main obstacles hinder effective learning: first, the limited availability of extracted teeth combined with crowded lab conditions often obscure demonstration details during live procedures, making it difficult to visualize complex three-dimensional tooth anatomy. Second, theoretical instruction often feels disconnected from practical application - especially for undergraduate students - as abstract explanations are hard to translate into clinical skills without hands-on reinforcement [5, 6]. Consequently, this disconnect makes it difficult for them to seamlessly transition from classroom learning to clinical practice. The use of digital technologies can clarify spatial relationships and promote procedural competence, better preparing students for clinical reality.

To bridge the gap between general virtual dental education and its application in prosthodontics, we focus this study on a specific and underrepresented area—inlay preparation training. With advancements in adhesive technology and minimally invasive restorations, inlay/onlay has emerged as a predominant approach for posterior tooth restoration, due to the advantage of effective preservation of remaining tooth [7]. In contrast to full crowns, inlays require more precise preparation parameters, such as margin design [8], cavity depth [9] and axial wall convergence [10], which are more difficult to teach and visualize using conventional methods. Improper tooth preparation can lead to adverse consequences including restoration defects, dislodgement, and tooth fracture [11]. Therefore, dentists’ proficient mastery of inlay tooth preparation techniques coupled with extensive practical experience are essential prerequisites for achieving ideal treatment outcomes. Despite their clinical importance, most existing digital simulation platforms emphasize full crown preparations [3, 12, 13], with limited focus on inlay training [14].

In response to this gap, we propose that establishing a digitized online learning platform would facilitate auxiliary training for dental preclinical students and enhance the quality of inlay restoration training significantly. Thus, the research was conducted to evaluate its impact on teaching quality and student satisfaction.

## Methods and materials

### Digital inlay training platform

**Fig. 1:**
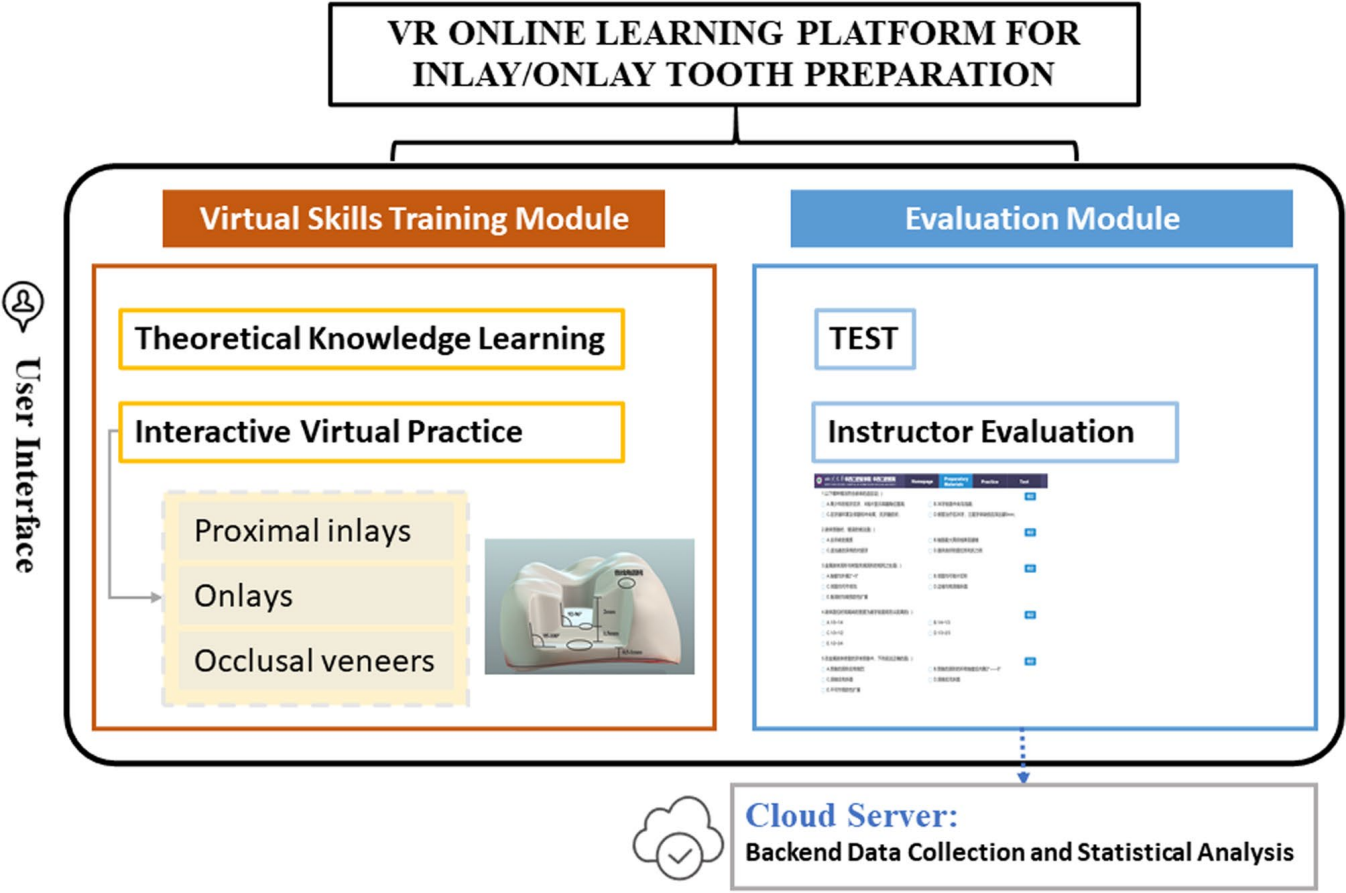
Schematic diagram of the VR platform’s architecture

**Fig. 2:**
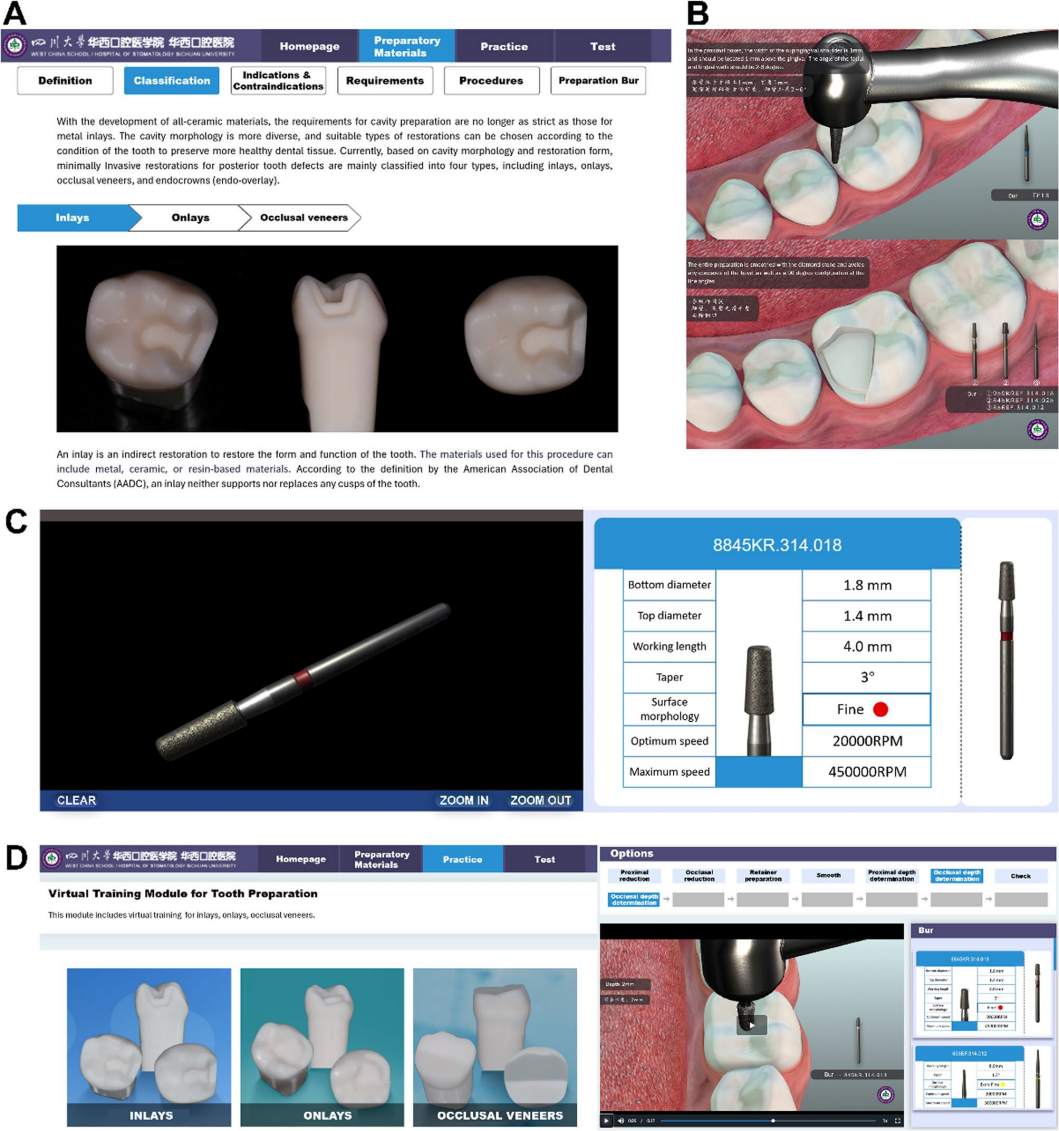
Primary steps of tooth preparation training in the digital platform for inlays. **A** the page for basic theoretical learning; (**B**) screenshots of instructional videos provided for users; (**C**) the page for the bur learning section; (**D**) the page for the virtual training module

We established a “Virtual Simulation Digital Online Learning Platform for Inlay Tooth Preparation,” developed by VIVEDM corporation (Chengdu, China). The platform was built using development tools including IntelliJ IDEA, Visual Studio, and Unity. The hardware environment used for development included an Intel i5-12400 processor, NVIDIA GeForce GTX 2060 graphics processing unit (GPU), 8 GB of random access memory (RAM), and 500 GB of available storage. The software operated on Windows 10, with Chrome browser compatibility for end users. This technology allowed students to interact with a three-dimension (3D) tooth model and simulate the preparation process using specialized virtual burs. The platform primarily consists of two modules: virtual skills training module, which includes theoretical knowledge learning and interactive virtual practice, and evaluation module **(**Fig. 1**)**. Students utilize this platform to practice inlay tooth preparation, including proximal inlays, onlays, and occlusal veneers. For example, the operational process for proximal inlays is briefly outlined. Figure 2 illustrates the primary steps of tooth preparation training. In addition, a demonstration video has been provided to briefly present the platform workflow (Supplementary File 1). Step 1 **(**Fig. 2A&B): Students access the platform, navigate to the theoretical section, review the standardized preparation process through graphics and text, and watch videos to reinforce their knowledge. Step 2 (Fig. 2C): They proceed to the bur learning section, where they familiarize themselves with the morphological characteristics and functional uses of specialized burs for inlays through the manipulation of a three-dimensional reconstructed bur model using a mouse. This lays a solid foundation for subsequent virtual simulation training. Step 3 (Fig. 2D): In the virtual training module, students are presented with a precisely modeled three-dimensional mandibular molar. They are required to select the sequence of tooth preparation steps and determine the relevant parameters for each step—including preparation depth and range, bur selection, retention form, resistance form, and key precautions. At each step, users must choose the correct parameter from four provided options in order to proceed to the next step. The platform also allows for detailed observation. Furthermore, each step is associated with relevant fundamental knowledge points for repeated learning and deeper understanding. Following self-paced online learning and virtual simulation operations, students, guided by instructors, return to offline classrooms to practice simulated tooth preparation on manikin heads.

### Participants

Participants were undergraduate dental students in their fourth to fifth years, who were in preclinical status, at Sichuan University West China School of Stomatology. They were predominantly male (40.36%) and female (59.64%), with an average age of 21.33 ± 0.90 years. To control for baseline knowledge and reduce potential confounding factors, students who had previously taken dental prosthodontics laboratory courses or attended lectures specifically on dental inlay restoration were excluded. These students were recruited through an on-campus student association. A total of 166 dental students voluntarily enrolled in this digital training program. Platform use was self-paced and unsupervised. Participants were required to use the digital training platform for a minimum of 30 min before the start of the formal course. They were instructed to complete all content on the platform before proceeding to the examination. This ensured that all participants had sufficient exposure to the training materials and the platform’s features prior to assessment.

### Survey questionnaire

At the conclusion of the training, participants completed a comprehensive examination, designed to evaluate their understanding of key restorative concepts—including inlay preparation techniques, material selection, preparation design, and treatment planning. Of the 166 enrolled students, 152 completed the assessment, yielding a dropout rate of 8.43%. We also employed detailed questionnaires to evaluate effectiveness, user satisfaction, and willingness to continue using the platform. Test and questionnaire were specifically developed for this study and has not been published previously. An English version was provided as a supplementary file (Supplementary File 2). The final test consisted of 12 questions with a maximum achievable score of 60, including various types of questions, such as multiple-choice, true/false, and multiple-response queries. These questions covered fundamental topics including tooth preparation principles for dental restorations, categorization of dental restorations, and procedural steps involved. This approach provided an objective assessment of students’ understanding of relevant knowledge after engaging with the online platform.

The secondary questionnaire was employed to gather comprehensive feedback from users regarding the effectiveness of implementing the virtual training platform in dental restorative experiments. This questionnaire aimed not only to collect basic demographic information but also to explore participants’ experiences with the platform, their study durations, and most importantly, their perceptions of the platform’s utility as a supplementary tool in fixed prosthodontics experiments. Prior to project commencement, we rigorously tested the questionnaire by inviting 10 students who were not part of the initial research cohort, ensuring that the questions were clear, unambiguous, and effectively captured necessary insights. The internal reliability of the questionnaire was assessed using Cronbach’s alpha, with satisfactory results (α = 0.959), indicating high reliability of the questionnaire for investigating satisfaction with platform use and teaching effectiveness. Following a pre-test phase, two experienced educators led group discussions aimed at refining and finalizing the questionnaire based on gathered feedback and insights to comprehensively capture required information. The questionnaire consisted of 13 distinct items; 12 of these utilized a 10-point Likert scale allowing participants to express views on a scale ranging from “0 = strongly disagree” to “9 = strongly agree”. Finally, an open-ended query concluded the questionnaire encouraging participants to provide suggestions for future evolution of online learning platforms and highlight any challenges or constraints encountered during clinical dental training.

The questionnaire items were initially drafted in Chinese to enhance comprehension among Chinese students. Participants voluntarily consented to participate in the study. All respondents were provided with comprehensive information regarding the research’s purpose and general procedures, along with a written consent form outlining study details. They were also informed about their right to withdraw from the study at any given time. Confidentiality was maintained by anonymizing data, with access restricted to the authors.

### Data analysis

The study data were processed using SPSS 26.0, which included internal reliability and validity tests as well as correlational analysis. The data obtained from the questionnaires were analyzed using Kaiser-Meyer-Olkin (KMO) measure and the Bartlett’s test of sphericity were employed to examine if the results met the prerequisite for factor analysis. To assess construct validity, confirmatory factor analysis (CFA) was performed on the 11 items excluding Q9, which was found to have a weak measurement relationship. Model fit was evaluated using several fit indices: comparative fit index (CFI), Tucker-Lewis index (TLI), and standardized root mean square residual (SRMR). Convergent validity was assessed through average variance extracted (AVE) and composite reliability (CR) values. The CFA supported a two-factor structure with satisfactory fit indices and reliability metrics. Then the correlations between each factor and the final student satisfaction was analyzed based on the linear regression model. Student satisfaction (dependent variable) was measured using Questionnaire Item 8: “Are you satisfied with the platform as a teaching aid in experiment course”. The independent variables were seven factors: interest, convenience, operational flexibility, academic standardization, organization of content, platform stability, and richness of course resources. We assessed multicollinearity using variance inflation factors (VIF). The independent variables were seven factors: interest, convenience, operational flexibility, academic standardization, organization of content, platform stability, and richness of course resources.

## Results

### Positive educational outcomes attributed to the inlay digital platform

**Fig. 3:**
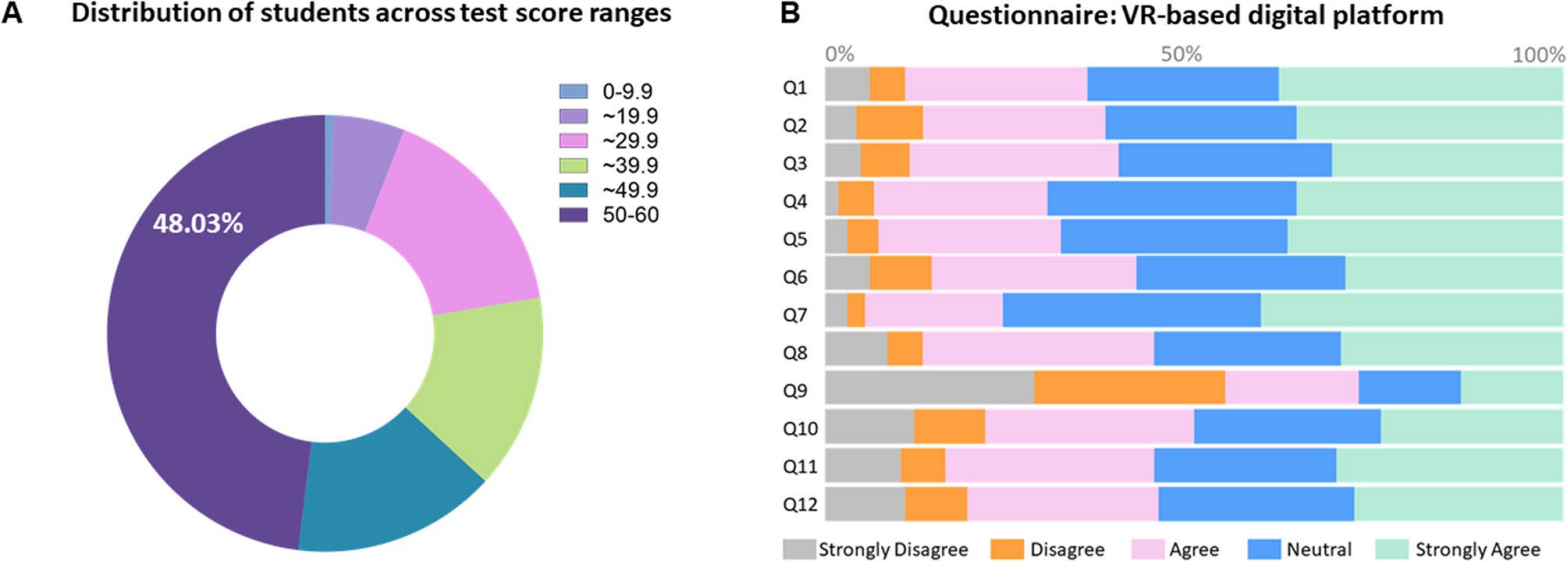
**A** Distribution of students across course test score ranges. The assessment consists of a total of 12 questions, with a maximum achievable score of 60; (**B**) Questionnaire feedback collected (*n* = 166)

A total of 91.57% of the students actively participated in the final course examination. The average score in the final exam was 43.32 ± 16.17 (maximum score: 60). Figure 3A depicts the distribution of the scores. The majority (63%) of participants demonstrated a basic understanding of the exam content by scoring between 30 and 50 points, indicating room for further improvement in application and mastery. Notably, 73 answer sheets scored or above 50, accounting for approximately 48.03% of all participants, highlighting their strong comprehension and proficiency in tooth preparation for dental restorations.

### High satisfaction with embedded platforms for preclinical teaching among students

The survey collected 166 valid questionnaires for 12 items. The results show that the overall user experience received high ratings (Table 1). Over 70% of users expressed satisfaction or high satisfaction with the platform (Fig. 3B), stating that the platform’s module settings are reasonable, its use is user-friendly, the operation is flexible, the system is stable, and the course resources are abundant. Most respondents believe the platform has increased their interest in independent learning and has been helpful in mastering fundamental knowledge related to dental restorations and improving independent operational skills. Some students believe that the platform can supplement offline practical teaching and replace some practical teaching hours. Over half of the users are willing to continue using the virtual simulation online platform in other chapters of practical teaching in dental restorations and would recommend it to their peers in related majors. However, a few students have suggested the need for further improvement and enrichment of course resources to enhance the platform’s educational assistance.

**Table 1:** Reliability assessment of the survey questionnaire

*N* of Items	*n*	Cronbach α
12	166	0.959

### Analysis of influencing factors of student satisfaction

Subsequently, exploratory factor analysis (EFA) was employed in order to extract crucial information from the data. Firstly, we analyzed whether the data is suitable for factor analysis (Table 2): the KMO measure yielded a value of 0.946, indicating that it met the prerequisite for factor analysis; furthermore, the Bartlett’s test of sphericity (*p* < 0.05) also confirmed that the research data was appropriate for factor analysis. Finally, three influencing factors were extracted: practicality, application prospects, and time consumption, with variance explanation rates of 42.81%, 31.35%, and 10.27%, respectively (Table 3). To clarify the meanings of the three factors extracted by exploratory factor analysis (Table 4), we provided representative items from the questionnaire for each category: (1) Practicality: This factor reflects users’ perception of whether the platform helped them practically in learning. For example, “The platform was helpful in mastering the key points of inlay tooth preparation.”; (2) Application Prospects: This refers to the perceived future use or valu e of the platform. For instance, “I am willing to continue using this platform in other prosthodontic training.” (3) Time Consumption: This factor relates to students’ perception of time efficiency. For example, “The time I spent on the platform was worthwhile and proportionate to the learning outcomes.” These examples aligned with the factor structure and helped to explain how students evaluated the platform from different perspectives. To further verify the construct validity of the questionnaire, we conducted CFA. Since Factor 3 (Time Consumption) contained only a single item, it could not be included in the CFA model. Therefore, CFA was performed on the remaining 11 items corresponding to two factors (Practicality and Application Prospects). The CFA results (Tables 5 and 6) indicated acceptable model fit (CFI = 0.941, TLI = 0.924, SRMR = 0.039), with all standardized loadings exceeding 0.83. Importantly, the corresponding AVE values were all greater than 0.50, and the CR values were all above 0.70, indicating good convergent validity of the measurement model (Table 7). These findings provide strong support for the validity and reliability of the two-factor model. Although all three factors could only explain 84.43% of the total variance, they comprised an effective index for evaluating the perceptions of the surveyed students (Table 4).

**Table 2:** KMO and Bartlett test

KMO		0.946
Bartlett test	Approx. Chi-Square	2179.604
	*df*	66
	*p* value	0.000**

*n* = 166

* p < 0.05

** p < 0.01

**Table 3:** The ratio of variance (Rotated)

Factor	Eigen	% of Variance	Cum. % of Variance
1	5.138	42.813	42.813
2	3.762	31.349	74.161
3	1.232	10.269	84.430

**Table 4:** Factor loading (Rotated)

Items	Factor loading			Communalities
	Factor 1	Factor 2	Factor 3	
5. Does the platform offer a wide range of course resources?	0.861			0.824
4. Are the settings for each section of the platform logically designed?	0.853			0.880
2. Is the platform user-friendly and easy to navigate?	0.798			0.827
3. How flexible is the platform in operation?	0.796			0.828
7. Are the course resources provided by the platform standardized in accordance with academic rigor?	0.760			0.795
1. Is the platform stable and reliable during usage?	0.726			0.740
6. Does using this platform for training provide an interesting experience?	0.698			0.794
8. Are you satisfied with using this platform as a teaching aid in dental restoration experiments?	0.525			0.843
12. Would you consider using a similar learning platform for other chapters, such as veneers, crowns, and active removable partial dentures?		0.824		0.916
10. Would you consider using this platform for self-study purposes?		0.795		0.909
11. Would you recommend this platform to fellow students majoring in dentistry at other universities?		0.784		0.782
9. Would you be willing to reduce offline training time by utilizing this platform?			0.950	0.993

* Rotation technique employed: Varimax, a method that aims to maximize variance

**Table 5:** Standardized factor loadings and squared multiple correlations

Factor	Item	Std. Loading	SMC
Factor1: Practicality	1. Is the platform stable and reliable during usage?	0.836	0.698
	2. Is the platform user-friendly and easy to navigate?	0.897	0.804
	3. How flexible is the platform in operation?	0.893	0.798
	4. Are the settings for each section of the platform logically designed?	0.906	0.820
	5. Does the platform offer a wide range of course resources?	0.837	0.701
	6. Does using this platform for training provide an interesting experience?	0.883	0.779
	7. Are the course resources provided by the platform standardized in accordance with academic rigor?	0.868	0.754
	8. Are you satisfied with using this platform as a teaching aid in dental restoration experiments?	0.854	0.730
Factor2: Application Prospects	10. Would you consider using this platform for self-study purposes?	0.942	0.887
	11. Would you recommend this platform to fellow students majoring in dentistry at other universities?	0.831	0.691
	12. Would you consider using a similar learning platform for other chapters, such as veneers, crowns and active removable partial dentures?	0.957	0.915

*Std. Estimate* Standardized Loading, *SMC* Squared Multiple Correlation

**Table 6:** Model fit indices

χ²/df	CFI	TLI	NFI	IFI	RMSEA (90% CI)	SRMR
3.932	0.941	0.924	0.923	0.941	0.133 (0.112–0.154)	0.039

*CFI* Comparative Fit Index, *TLI* Tucker–Lewis Index, *NFI* Normed Fit Index, *IFI* Incremental Fit Index, *RMSEA (90% CI)* Root Mean Square Error of Approximation, *SRMR* Standardized Root Mean Square Residual

**Table 7:** Convergent validity

Factor	AVE	CR
Practicality	0.761	0.962
Application Prospects	0.831	0.936

*AVE* Average Variance Extracted, *CR* Composite Reliability

To further investigate the factors impacting teaching satisfaction, we conducted a multiple linear regression analysis. Table 8 presents the correlations between each factor and the final student satisfaction. One item showed VIF value slightly above 5, while all others were below 5. As these values are well below the threshold of 10, multicollinearity was considered moderate and not severe, and all predictors were retained in the regression model. The findings revealed that stability in embedded digital platform usage (regression coefficient = 0.207, *p* < 0.01), interest (regression coefficient = 0.577, *p* < 0.01), and academic standardization of learning resources (regression coefficient = 0.201, *p* < 0.05) significantly and positively influence student satisfaction. However, convenience, operational flexibility, section organization, and richness of course resources did not exert a significant impact on student satisfaction.

**Table 8:** Parameter estimation of linear regression

	Unstandardized Coefficients		Standardized Coefficients	t	*p*	Collinearity Diagonostics	
	B	Std. Error	Beta			VIF	Tolerance
Constant	−0.056	0.224	-	−0.252	0.802	-	-
1. Is the platform stable and reliable during usage?	0.207	0.074	0.201	2.791	0.006**	3.093	0.323
2. Is the platform user-friendly and easy to navigate?	0.064	0.095	0.061	0.671	0.503	4.958	0.202
3. How flexible is the platform in operation?	0.029	0.096	0.026	0.297	0.767	4.747	0.211
4. Are the settings for each section of the platform logically designed?	0.023	0.120	0.019	0.190	0.849	5.669	0.176
5. Does the platform offer a wide range of course resources?	−0.123	0.092	−0.106	−1.343	0.181	3.693	0.271
6. Does using this platform for training provide an interesting experience?	0.577	0.079	0.558	7.290	0.000**	3.498	0.286
7. Are the course resources provided by the platform standardized in accordance with academic rigor?	0.201	0.098	0.164	2.058	0.041*	3.802	0.263
*R* ^2^	0.735						
Adj *R* ^2^	0.724						
*F*	*F* (7,158) = 62.705,*p* = 0.000						
D-W	2.048						
** *p* < 0.01							

Dependent Variable: 8. Are you satisfied with using this platform as a teaching aid in dental restoration experiments?

*n* = 166

* *p* < 0.05

** p<0.01

### Valuable recommendations from trainees for the inlay digital platform

A multitude of students shared their experiences and suggestions for further optimizing the platform. The prevailing consensus among students is that the current academic standard of course resources is commendable, with an expressed desire for augmented learning content pertaining to case discussions. Some students identified issues such as system lags, poor compatibility with operating environments, and an unattractive interface design. They aspire to enhance system compatibility and responsiveness, introduce scoring and login features for improved tracking and personalization, as well as streamline the interface to minimize scrolling and bolster operability. The widespread implementation of this digital platform in dental restoration experiments is eagerly anticipated by many students. However, some students contend that online platforms cannot entirely replace practical hands-on experience. To address this concern, these students have suggested incorporating virtual simulation modules with tactile feedback systems due to their pivotal role in augmenting clinical skills.

## Discussion

Tooth preparation for inlay/onlay is one of the essential basic skills for dental students. Traditionally, dental clinical skill training primarily around the world relies on extracted teeth, and artificial teeth [15]. However, the supply of extracted teeth is limited, and artificial teeth are not only costly but also structurally and materially different from natural teeth, which limits the scope of training programs. Consequently, many students feel unfamiliar with operative procedures when they start the medical internship, which may lead to doctor-patient disputes and reducing their confidence and enthusiasm [16, 17].

**Fig. 4:**
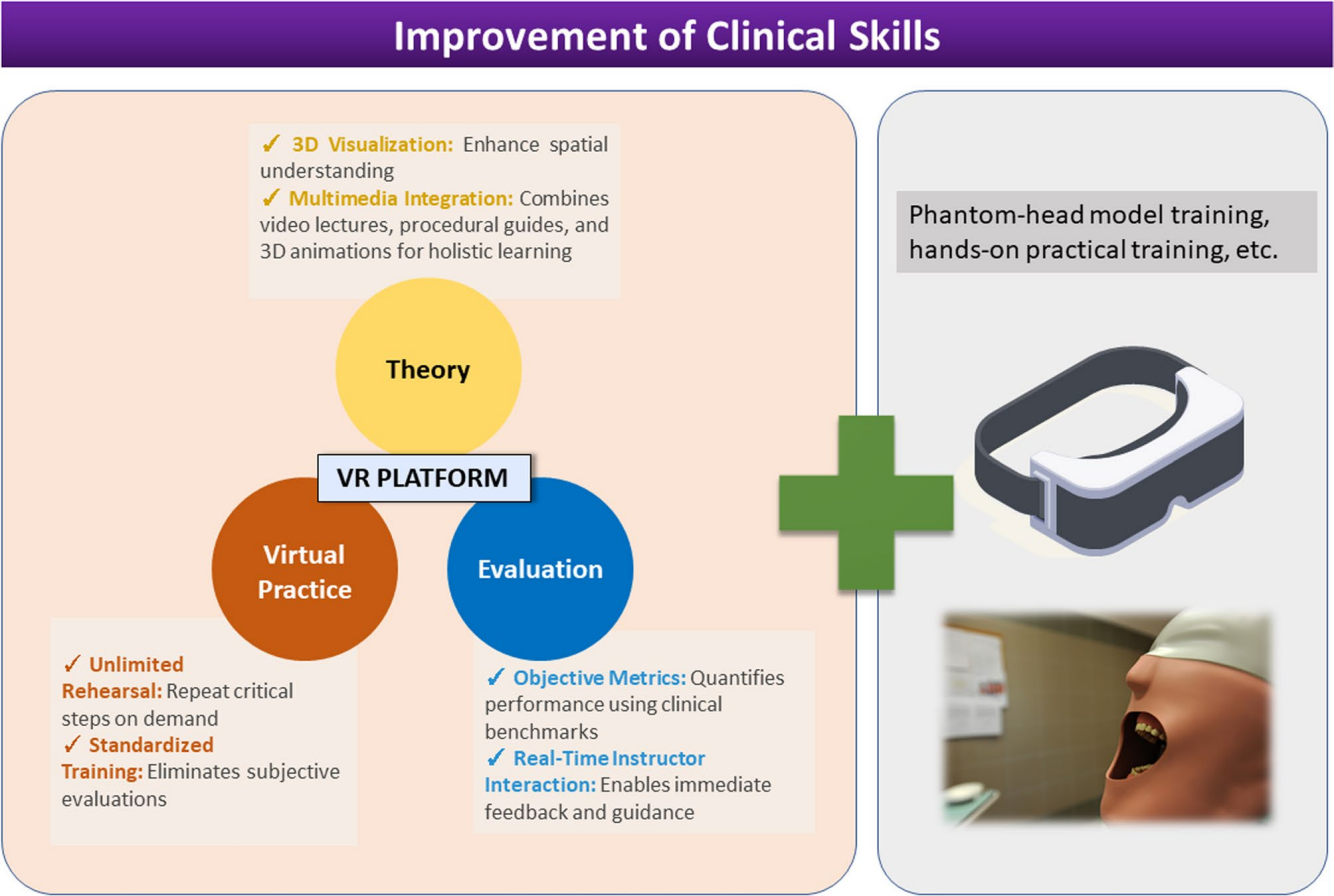
Advantages and function of the VR platform. The digital platform includes three core modules: theoretical learning (3D visualization, multimedia resources), virtual practice (repetitive rehearsal, interactive sequencing), and evaluation (objective metrics, instructor feedback). Combined with phantom-head simulation, this model enhances clinical skill development

Recently the application of digital oral simulators based on VR technology has made significant progress in dental practice training, covering procedures such as tooth preparation [18], implantation [19], tooth extraction [20], maxillofacial surgical procedures [21], periodontal therapy [22], and orthodontics [23]. In our study, we utilized digital technology for 3D modeling and incorporated rich multimedia resources to construct a virtual experimental network platform specifically designed for inlay/onlay tooth preparations. (Fig. 4). Through this platform, users can learn theoretical concepts and gain familiarity with various clinical operational procedures for tooth preparation by using commonly used burs and instruments through interactive virtual experimental practice. Furthermore, procedural sequencing steps, which enable the operator to determine the sequence of clinical procedures can further help students to cultivate a comprehensive clinical mindset. Traditional evaluations rely on teacher observation, which may overlook errors and demands high faculty involvement. Conversely, virtual simulation practical teaching platforms can standardize every operational step and detail using 3D virtual technology, quantifying adjustments based on student operations and procedural sequences. This approach presents a more objective and specific final effect of practical teaching. Our results demonstrate that incorporating the digital platform into preclinical skills training significantly enhances teaching effectiveness. Most students achieved good grades after using the platform. However, when faced with questions closely aligned with clinical scenarios, there was a relatively higher error rate observed, indicating the need for further improvement during subsequent clinical internships.

Compared with existing studies, our findings show similar high student satisfaction. For instance, Felszeghy [18] reported that students gave highly positive evaluations of a VR-haptics simulator for improving manual dexterity. While our platform lacks haptic feedback, students appreciate its convenience and flexibility, as it can be used anytime and anywhere without physical space constraints. Future upgrades will include cloud-based access to haptic systems to combine the benefits of immersive realism with flexible learning.

Our results demonstrate that incorporating the digital platform into preclinical skills training significantly enhances teaching effectiveness. While the average score was 43.32 out of 60, suggesting a moderate level of achievement, a significant proportion of students scored below 50. Several factors may explain this disparity. First, student engagement time was not standardized or monitored, leading to possible variation in preparation levels. Second, the absence of haptic feedback may have reduced motivation for some learners, thereby affecting practice quality. Additionally, the platform focuses on procedural simulation, so students’ attention is directed toward stepwise workflow, whereas the course assessment included several clinically oriented items; this mismatch likely contributed to lower scores on some questions. These findings point to the need for enhanced interactivity, performance feedback, and structured learning scaffolds in future platform development. To better support students who underperform, we implemented several measures: (1) maintaining open access to the platform post-study to encourage independent practice; (2) offering ongoing complementary offline clinical skills courses. These strategies are designed to offer more personalized and effective support, enabling students who require additional practice time to enhance their competencies.

Furthermore, it is important to note that while the 3D digital platform has shown significant promise in preclinical dental education, continued development and system enhancement are necessary. Student feedback pointed out the lack of haptic feedback, which is widely considered important for developing tactile perception and fine motor skills [24]. Students are unable to practice depth control and margin refinement through the current platform. Although the system does not yet support tactile training due to cost constraints, previous studies suggest that VR practice without haptics may allow learners to focus more on procedural logic and theoretical knowledge, potentially improving later hands-on efficiency [25]. We recognize the importance of haptic feedback for comprehensive skill development. As part of our future work, we are actively working on integrating a cloud-based haptic feedback system into the platform to enhance the realism and effectiveness of the training experience. Additionally, the single-institution study design and short-term evaluation limit the generalizability and long-term applicability of our findings. Longitudinal studies are needed to assess skill retention and clinical transferability. In the construct validity analysis, the “Time Consumption” factor was represented by only a single item, preventing its inclusion in the CFA. Future versions of the questionnaire will incorporate multiple items to better capture students’ perceptions of efficiency. Finally, the absence of a control group limits the strength of our claims regarding the VR platform’s effectiveness compared to traditional methods. Future studies should include a control group for a more robust evaluation.

## Conclusion

We have established an online teaching platform focusing on digitalized fixed prosthodontic tooth preparation. After the application of the digital teaching platform for inlays, we significantly improved the quality of experimental teaching in dental restoration, exploring a standardized new approach for practical teaching in tooth preparation. In the future, it should be optimized through software design upgrades, combining tactile feedback training modules, and covering more chapters of prosthodontics.

## Supplementary Information

Supplementary Material 1

Supplementary Material 2

## Data Availability

The datasets generated and/or analyzed during the current study are not publicly available due to the protection of student privacy but are available from the corresponding author on reasonable request.

## Abbreviations

VR: Virtual Reality
AR: Augmented Reality
3D: Three-Dimension
KMO: Kaiser-Meyer-Olkin
CFA: Confirmatory Factor Analysis
VIF: Variance Inflation Factors
EFA: Exploratory Factor Analysis
CFI: Comparative Fit Index
TLI: Tucker-Lewis Index
SRMR: Standardized Root Mean Square Residual
AVE: Average Variance Extracted
CR: Composite Reliability
