# Supplementary material for: Enhancing preclinical dental education through a VR-based digital platform for inlay tooth preparation
Source: BMC Med Educ. 2025 Nov 21;25:1630. doi: 10.1186/s12909-025-08138-y (PMC12639650; doi:10.1186/s12909-025-08138-y)
Supplement: Supplementary file 1 — Supplementary Material 1 [file 12909_2025_8138_MOESM1_ESM.pdf]

TEST For Inlay

Single-choice Questions

Answer

1.Which of the following is **NOT** an advantage of inlay restorations?

2.After root-canal treatment on tooth #37, there is a mesial wall defect, with the remaining axial walls ≥ 2 mm thick and a clinical crown height of 4 mm. The patient requests the most minimally invasive option. Which of the following restorations is most appropriate?

3.The indications for inlay restorations include:

4.Regarding occlusal-surface tooth preparation for ceramic inlays, which of the following is correct?

Multiple-choice Questions

1.The tooth-preparation requirements for ceramic inlays include:

2.Regarding tooth preparation for high-strength ceramic inlays, which of the following is correct?

3.Regarding proximal-surface tooth preparation for metal inlays, which of the following is correct?

True/False Questions

1.The margins of an inlay's occlusal surface can be placed within the functional occlusal contact areas.

2.Ceramic inlays can gain additional retention by preparing slot-like proximal surfaces.

3.When the occlusal defect is extensive (e.g., a cusp fracture), a high-strength ceramic inlay should be considered.

4.For tooth #16 with mesial and occlusal caries—where the opposing tooth is restored with a palladium-silver alloy crown—a gold-alloy inlay on #16 may be indicated.

5.During inlay tooth preparation, the divergence angle of the axial walls must not exceed 6°.

Survey On VR Platform For Assisting Inlay Tooth Preparation Teaching

Instructions: Please rate your satisfaction or feeling with the virtual reality platform on a scale of 0 (Strongly Disagree) to 9 (Strongly Agree).

Questions:	Rating scale: Strongly Disagree	Disagree	Neutral	Agree	Strongly Agree
1. Is the platform stable and reliable during usage?	☐	☐	☐	☐	☐
2. Is the platform user-friendly and easy to navigate?	☐	☐	☐	☐	☐
3. How flexible is the platform in operation?	☐	☐	☐	☐	☐
4. Are the settings for each section of the platform logically designed?	☐	☐	☐	☐	☐
5. Does the platform offer a wide range of course resources?	☐	☐	☐	☐	☐
6. Does using this platform for training provide an interesting experience?	☐	☐	☐	☐	☐
7. Are the course resources standardized in accordance with academic rigor?	☐	☐	☐	☐	☐
8. Are you satisfied with the platform as a teaching aid in experiment course?	☐	☐	☐	☐	☐
9. Would you be willing to reduce offline training time by utilizing this platform?	☐	☐	☐	☐	☐
10. Would you consider using this platform for self-study purposes?	☐	☐	☐	☐	☐
11. Would you recommend this platform to fellow students at other universities?	☐	☐	☐	☐	☐
12. Would you consider using a similar learning platfrom for other chapters?	☐	☐	☐	☐	☐
